# Identification of Achille’s Tendon Tears: Diagnostic Accuracy of Dual-Energy CT with Respect to MRI

**DOI:** 10.3390/jcm13154426

**Published:** 2024-07-29

**Authors:** Giovanni Foti, Luca Bortoli, Matteo Tronu, Sabrina Montefusco, Gerardo Serra, Roberto Filippini, Venanzio Iacono

**Affiliations:** 1 Department of Radiology, IRCCS Sacro Cuore Hospital, 37042 Negrar, Italy; 2 Department of Radiology, Verona University Hospital, 37126 Verona, Italy; lbortoli95@gmail.com (L.B.); matteo.tronu.94@gmail.com (M.T.);; 3 Department of Anesthesia and Analgesic Therapy, IRCCS Sacro Cuore Don Calabria Hospital, 37024 Negrar, Italy; gerardo.serra@sacrocuore.it; 4 Department of Sports Medicine, IRCCS Sacro Cuore Hospital, 37042 Negrar, Italy; roberto.filippini@sacrocuore.it; 5 Department of Orthopaedics, IRCCS Ospedale Sacro Cuore Don Calabria, 37024 Negrar, Italy; venanzio.iacono@sacrocuore.it

**Keywords:** Achilles tendon, dual-energy CT, MRI, tear, rupture

## Abstract

**Background**: The aim was to assess the diagnostic accuracy of DECT in diagnosing Achilles tendon tears, using MRI as the reference for diagnosis. **Methods**: This feasibility study conducted prospectively at a single center included consecutive patients suffering from ankle pain who underwent DECT and MRI between April 2023 and October 2023. A total of three radiologists, blinded to the patient’s clinical data, assessed the images. Achille Tendon injuries were diagnosed in case of thickened and inflamed tendons or in case of a partial or complete tear. Diagnostic accuracy values of DECT were calculated using a multi-reader approach. Inter-observer agreement was calculated using k statistics. **Results**: The final study population included 22 patients (mean age 48.5 years). At MRI, Achille’s tendon lesion was present in 12 cases (54.5%) with 2 cases of complete rupture, 8 cases of partial tear (5 with tendon retraction), and 2 cases of tendon thickening. The mean thickness of injured tendons was 10 mm. At DECT, R1 was allowed to correctly classify 20/22 cases (90.9%), R2 19/22 cases (86.4%), and R3 18/22 cases (81.8%). At DECT, the mean thickness of the positively scored tendon was 10 mm for R1, 10.2 mm for R2, and 9.8 mm for R3. A very good agreement was achieved with regard to the evaluation of tears (k = 0.94), thickness (k = 0.96), and inflammatory changes (k = 0.82). Overall agreement was very good (k = 0.88). **Conclusions**: DECT showed a good diagnostic performance in identifying Achille’s tendon tears, with respect to MRI.

## 1. Introduction

The Achilles tendinopathies represent a relatively common condition in clinical practice [1]. There are varied cases, ranging from mild tendon discomfort and functional impairment to more or less profound tears, culminating in complete ruptures [2]. While patients without tears can be managed with a conservative, medical, or functional approach, in cases of tears, surgical therapy might be necessary [3,4,5]. Furthermore, the type of surgical approach can vary from open surgery to minimally invasive or percutaneous, depending on the surgeon’s choice and the clinical situation of each individual patient [6]. Moreover, an early diagnosis of an initial tear could improve the patient’s prognosis, reducing the risk of ruptures or ensuring better chances of healing within relatively short periods [3,4,5,6].

Among the most used methods for evaluating the Achilles tendon, ultrasound is certainly the most used method [7,8]. This method has the advantage of high spatial resolution capable of identifying tendon thickening or small tendon tears and evaluating the presence of inflammation using Doppler imaging [8]. However, it is highly dependent on the operator. Moreover, this method can sometimes be unreliable in preoperative planning, as it may not identify the causes of ankle pain, and notably, it does not allow for bone assessment.

Magnetic resonance imaging (MRI) represents a largely used method for preoperative assessment of Achilles tendon tears [9,10,11,12]. This method allows for the evaluation of other causes of ankle pain, including areas of bone edema, osteochondral lesions of the talus, instability, and soft tissue injuries. However, MRI is not always available due to its high costs and the possibility of absolute and relative contraindications.

Dual-energy CT (DECT) has been extensively utilized in musculoskeletal imaging, providing the ability to achieve high tissue contrast in soft tissues, enhanced by specific applications, and high-resolution images with bone filters [13,14,15,16,17]. For preoperative evaluation of the Achilles tendon, this method can reveal tendon inflammation, thickening, partial tears, or complete ruptures [18,19,20]. Additionally, it can exclude other lesions and show signs of posterior impingement, such as bone edema at the heel [21,22,23,24,25,26,27]. Moreover, DECT can highlight the prominent appearance of the posterior–superior margin of the heel bone, which could be a potential cause of tendon pathology. Finally, it can display partial calcification of the Achilles tendon and plantar fascia, as well as potential bone spurs.

A report exists that showcases the effective use of DECT in studying the Achilles tendon for diagnosing tears [28]. However, as far as the author is aware, there is currently no reliable data in the literature that compare the diagnostic accuracy of this method to MRI. In this context, DECT could serve as an alternative to MRI for diagnosing structural lesions of the Achilles tendon and planning surgical interventions for patients with contraindications to MRI.

For this reason, the purpose of our study was to assess the diagnostic accuracy of DECT in diagnosing Achilles tendon tears, using MRI as the reference for diagnosis.

## 2. Methods

### 2.1. Participants

This prospective feasibility single-center study was carried out from April 2022 to November 2023. The study received approval from the institutional review board (IRB—CESC protocol. 3762), and written informed consent was obtained from all participants. We enrolled patients suffering from ankle foot pain who underwent both MRI and DECT examinations (interval range 1–6 days). Exclusion criteria included insufficient quality of any imaging tests, metallic hardware, or previous ankle surgery. All patients with other pathological findings that met the inclusion criteria for another parallel study were excluded, such as those with clinical suspicion of foot stress fractures. This decision was made to avoid the risk of memory bias.

Patients were referred to the radiology department following a dedicated specialistic visit with an orthopedic surgeon because of ankle pain. 

### 2.2. Magnetic Resonance Imaging

The MRI scans were conducted using a 3 Tesla Scanner (Omega, United Imaging, Shanghai, China) using multiplanar T1, T2, and PD Fat-saturated sequences. The protocol with detailed imaging parameters is summarized in Table 1. 

The PDw FSE FS is acquired in all orientations (coronal, sagittal, and transverse). The T1w FSE is acquired in the coronal and sagittal plane, while the T2w FSE is acquired in the transverse plane: T1w FSE, T1-weighted Fast Spin Echo; T2w FSE, T2-weighted Fast Spin Echo; PDw FSE FS, PD-weighted Fast Spin Echo Fat Sat.

### 2.3. DECT Protocol

The dual-energy CT scans were conducted without the administration of contrast material intravenously. We used a dual-source scanner (Somatom Definition Force, Siemens Healthineers, Forchheim, Germany). Tube voltages were set at 80 and 150 kVp with a tin filter. The tube current–time product was adjusted at 1.6:1 (tube A, 220 mAs; tube B, 138 quality reference mAs). Through the incorporation of automated attenuation-based tube current modulation (CARE dose 4D), a radiation burden similar to that of comparable previous studies was maintained. Patients were imaged supine with the affected foot positioned at the center of the gantry. The unaffected foot and leg were bent near the contralateral knee to avoid beam-hardening artifacts.

### 2.4. DECT Post-Processing

The images obtained at 80 kV and 150 kV with the soft-tissue kernel (Qr32), at a thickness of 0.75 mm and an increment of 0.6 mm, were transferred to a dedicated PC (SyngoVia^®^ VB40, Siemens, Erlangen, Germany). The Virtual Non-Contrast (VNC) application with a fat map was used to create specific maps for tendon and ligament evaluation. Normal tendons and ligaments were visualized on color-coded maps overlaid on gray-scale CT images within the postprocessing software (1 mm reconstruction). Specifically, tendons and ligaments were coded in blue-scale colors, while yellow superimposed images indicated the presence of tears and/or inflammatory changes. For each participant, isotropic image datasets using soft tissue and bone window settings across the preferred imaging planes were available for clinical reading.

### 2.5. Image Analysis

MRI images, representing the ground truth for this feasibility study, were assessed in consensus by two experienced MSK radiologists (LR and EO with 27 and 12 years of experience, respectively). On MRI, the diagnosis of an Achilles tendon lesion was defined as follows: Diagnosis of tendinopathy was made in cases of increased tendon thickness (>8 mm), with or without increased signal intensity on T2-weighted images, without tendon tear. The presence of peri-tendinous inflammation, indicated by fluid or edema, was noted. Diagnosis of a tear was made if there was a loss of tendon substance (measured in mm), with partial tendon involvement, with or without partial tendon retraction (measured in mm). A rupture was defined by the presence of a complete laceration of the tendon, with clear retraction of the majority of tendon fibers.

DECT images, representing the test images, were evaluated independently by three radiologists with varying levels of experience (GF with 17 years of experience, and two residents with 1 and 4 years of experience in MSK radiology). The radiologists were blinded to the clinical and MRI findings. On DECT, the diagnosis of tendinopathy was based on the presence of increased tendon thickness (>8 mm), with or without increased water content on DECT superimposed images, without tendon tear. The presence of inflammatory changes, diagnosed by fluid or edema around the tendon, was noted. Diagnosis of a tear was made if there was a loss of tendon substance (thickness of the tear measured in mm on the axial plane), with partial tendon involvement, with or without partial tendon retraction (length measured in mm on the sagittal plane). A rupture was defined as a complete laceration of the tendon, with clear retraction of the majority of tendon fibers. Additional imaging parameters, including tendon calcifications, posterior calcaneal spur, Haglund’s deformity, and calcaneal edema, were recorded.

A binary approach, distinguishing the presence versus the absence of a lesion, was employed for reading and subsequent statistical analysis.

### 2.6. Statistical Analysis

For this feasibility study, a sample size calculation was not performed, and a convenient sample of consecutive participants was enrolled. To perform an assessment of DECT capability in diagnosing Achille’s tendon changes, MRI images were considered as a reference standard for diagnosing (consensus reading of 2 experienced MSK radiologists).

At MRI, all patients with any tendon changes, including thickening or partial or complete tears, were scored as positive, whereas patients with normal tendon appearance at MRI were scored as normal and were considered as case–control. 

Demographic and clinical data were summarized using descriptive statistics and measures of variability, reported with 95% confidence intervals. Sensitivity and specificity were calculated using a binary approach (distinguishing positive versus negative cases). The interobserver agreement was assessed using the K index. A value of *p* < 0.05 was considered significant. Statistical analysis was performed by the same authors.

## 3. Results

### 3.1. Patients Results

Three patients were excluded from the study because of previous tendon surgery (*n* = 1), previous ankle surgery with subsequent artifacts (*n* = 1), and because of poor MRI quality caused by excessive motion artifacts (*n* = 1).

**Figure 1:**
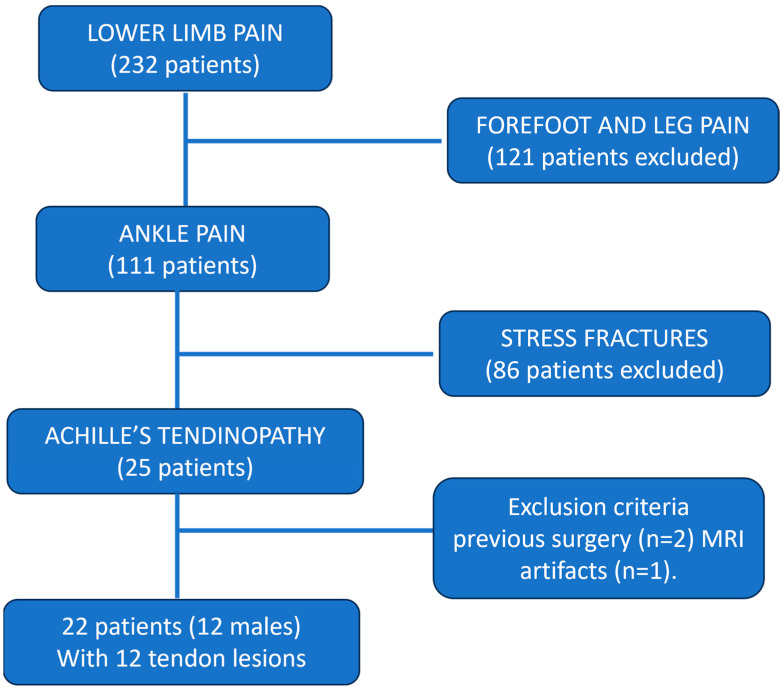
Diagram showing patient flowchart with exclusion criteria.

The patient’s cohort included 12 males and 10 females. The mean age was 48.5 years, range 26–78 years. There was no significant difference between positive and negative cases concerning sex and mean age (*p* > 0.05). A total of 8 patients were studied due to recent trauma, 6 among those with a tear. Because of a lack of detailed clinical or surgical data, we cannot define if a pre-existent tendinopathy was present before the acute trauma. Demographic features of patients enrolled are summarized in Table 2. Figure 1 represents a diagram showing a patient flowchart with exclusion criteria.

### 3.2. MRI Results

At MRI, 10 patients presented regular signals and thickness of tendon, without a partial or complete tear.

The presence of Achille’s tendon lesion was present in 12 cases (54.5%) with 2 cases of complete rupture, 8 cases of partial tear (5 with tendon retraction), and 2 cases of tendon thickening without tear. The thickness of a partial tear ranged from 1 mm to 8 mm (mean 3.8 mm), whereas the length of retraction ranged from 2 mm to 34 mm (mean 16.2 mm). Also, clear inflammatory changes in adjacent tissues were depicted in 7 cases, two without tendon lesions. 

The mean thickness of injured tendons was 10 mm, whereas that of normal tendons was 6.5 mm. 

### 3.3. DECT Results

The results of the DECT reading sessions of the three readers involved in the study are summarized in Table 3.

With regard to tendon lesions, R1 was allowed to correctly classify 20/22 cases (90.9%), missing a subtle partial tear (in a not particularly thickened and inflamed tendon), and pointing out a false-positive (FP) partial tear. R2 was allowed to correctly classify 19/22 cases (86.4%), missing the same partial tear as R1 and a case of an inflamed and thickened tendon, and pointing out the same FP (incorrectly scored as partial tear) as the R1. Finally, R3 was allowed to correctly classify 18/22 cases (81.8%), missing the same partial tear as R1 and R2, and pointing out 2 cases of FP inflamed and thickened tendon, without pointing out false-positive tears. 

Both the complete tears were detected and classified correctly at DECT by the three readers. A subtle partial tear was missed by all three readers. 

At DECT, the mean thickness of the positively scored tendon was 10 mm for R1, 10.2 mm for R2, and 9.8 mm for R3. The mean thickness of the negatively scored tendon was 7 mm for R1, 6.8 mm for R2, and 7.3 mm for R3. 

**Figure 2:**
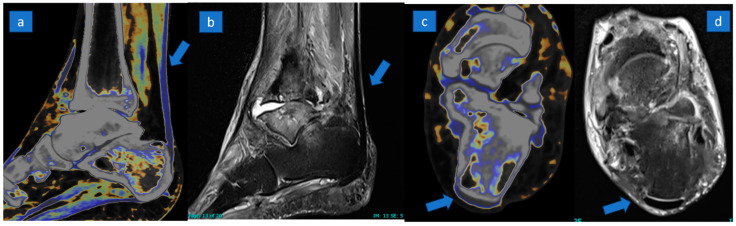
A 57-year-old male with a painful ankle due to rheumatoid arthritis. Normal tendon (arrow) is coded in blue on sagittal (**a**) and axial (**c**) super-imposed color-coded maps. The normal appearance of a tendon (arrow) is confirmed on sagittal (**b**) and axial (**d**) PD Fat-saturated MR images.

**Figure 3:**
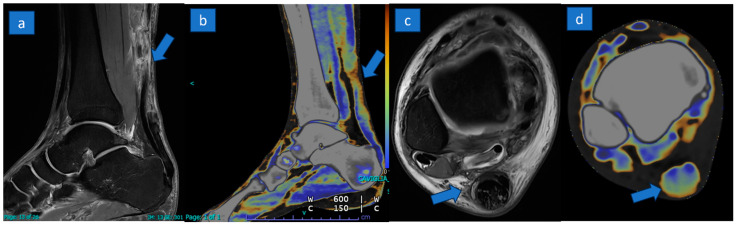
A 27-year-old professional soccer player with a complete rupture of the proximal tendon. A complete tear with tendon retraction (arrow) is depicted on sagittal PD Fat-saturated MR image (**a**) and confirmed on the corresponding sagittal reconstructed DECT super-imposed color-coded image (**b**). Severe tendon thickening (arrow) with mild inflammatory changes is recognizable on the distal aspect with a good correlation between MRI (**c**) and DECT (**d**) axial images.

**Figure 4:**
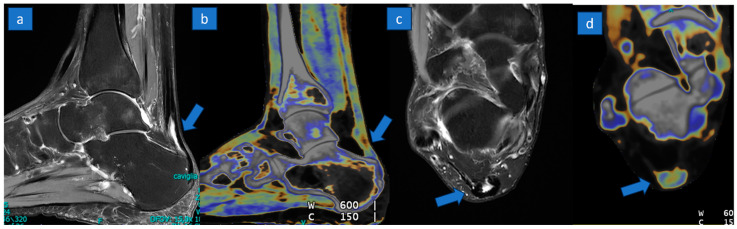
A 55-year-old woman with a partial tear of the distal aspect of Achille’s tendon. A partial tear with partial tendon retraction (arrow) is depicted on sagittal PD Fat-saturated MR image (**a**); on the corresponding sagittal reconstructed DECT super-imposed color-coded image (**b**) there is the loss of normal tendon appearance, but it is not possible to rule out a complete tear. The partial, horseshoe-shaped anterior tear (arrow) is recognizable on the distal aspect with a very good correlation between MRI (**c**) and DECT (**d**) axial images.

Explicative comparative cases of normal tendon, partial tear, and complete tear are shown in Figure 2, Figure 3 and Figure 4.

### 3.4. DECT Inter-Observer Agreement

A very good agreement was achieved between the three readers with regard to the evaluation of tears (k = 0.94), tendon thickness (k = 0.96), and tendon inflammatory changes (k = 0.82). Overall agreement was very good (k = 0.88). Agreement values are summarized in Table 4.

## 4. Discussion

In this feasibility study, the possibility of diagnosing structural injuries and inflammatory changes in the Achilles tendon using DECT in a limited patient cohort was assessed. For this purpose, MRI was employed as the diagnostic standard to closely simulate clinical practice. Alongside evaluating cases with complete tears, easily identifiable via DECT due to significant tendon lacerations and retractions, the presence of partial tears, and peri-tendinous inflammatory alterations were examined. The preliminary findings of this study have confirmed the significant potential of DECT in assessing the Achilles tendon. Specifically, the diagnostic accuracy values ranged from 90.9% for reader 1 to 81.8% for reader 3. These differences are likely largely attributed to varying levels of experience among readers, not only in musculoskeletal imaging but especially in the application of DECT techniques in the musculoskeletal field. As anticipated, reader 1, who is more experienced, achieved better results. Nevertheless, the inter-observer variability was consistently good for all evaluated parameters, affirming the excellent capabilities offered by this technique.

In fact, the possibility of using DECT for diagnosing Achilles tendon lesions had already been proposed in a study dating back to 2013 [28]. In said study, authors described a case of Achilles’ tendon tendinopathy accompanied by an initial identification of a partial tear via DECT utilizing a collagen material decomposition tool. Although we agree that MRI maintains its superiority in terms of image precision for detecting tendinopathy in the ankle, we believe there is potential for increased utilization of DECT in tendon assessment. This applies both to incidental evaluations during exams performed for other reasons in various body regions and to the growing elderly population. DECT could be particularly useful for follow-up of known injuries, as it avoids the issue of radiation exposure and can save time and reduce costs. Additionally, DECT stands out by providing supplementary soft tissue data beyond what traditional CT scans offer [29]. Previous studies that focused on the accuracy of DECT in assessing ligamentous structures primarily examine the anterior cruciate ligament in the knee, showing very good diagnostic accuracy values, with respect to MRI [30,31,32,33]. These values are consistent with those found in our study. However, it is noteworthy that few studies concentrate on tendon evaluations. Moreover, these studies employed specific applications tailored for the structures under examination (collagen tool), whereas we adapted an application originally designed for abdominal use, specifically targeting fat mapping. The rationale behind this off-label use is that tendinous structures, particularly the Achilles tendon, are typically surrounded by adipose tissue (as seen in the Kager triangle), which naturally contrasts with the normal tendon structure (rich in collagen). The increased water content in cases of inflammation raises density relative to fat, which usually characterizes the inflammatory process. Suboptimal results might be due to the inclusion of cases presenting inflammation without structural lesions. However, in clinical practice, encountering such cases is common, hence the necessity to explore the potential extent to which DECT could be effective in addressing these scenarios. Another important aspect to consider is the presence of false positives, which are indeed present. The application is not specifically tailored, and even mild thickness variations or subtle density alterations might simulate minor lacerations or inflammation. It is important to highlight that DECT has, however, identified complete or partial tendon tears with retraction. In chronic rupture, DECT can reliably demonstrate fatty replacement in the area of muscle and tendon tear.

DECT likely represents one of the most powerful and reliable imaging techniques for evaluating gout-related lesions, which can also affect the Achilles tendon [30,31]. Furthermore, DECT has been successfully used to identify bone bruises in the rear foot [24,25,26,27]. In the latter case, DECT offers the undeniable advantage of not only providing dedicated maps and applications for assessing edema or specific inflammatory pathologies but also ensuring high-resolution images for bone evaluation, both in two and three dimensions. In this context, DECT could be an excellent method for surgical planning in morphological alterations of the hindfoot, such as Haglund’s deformity or clubfoot. Additionally, DECT might better highlight the presence of bone fragments or tendon calcifications compared to MRI. 

Imaging post-surgery allows the study of the intrinsic characteristics of tendon fibers. Follow-up of an operated tendon is clinical, but MRI or US may give important information regarding general morphology and tendon structure. In particular, US plays a crucial role in the follow-up of operated tendons because of the dynamic nature of this technique and the contribution of the color-doppler tool, and MRI has shown to be a useful method to evaluate the healing process of surgically treated Achilles tendon [6]. In this scenario, CT could represent a valid alternative to MRI or ultrasound, especially when the intervention also involves the contiguous bone components.

This study has several limitations. Firstly, few patients were enrolled. However, considering that it was a feasibility study exploring a new application for DECT, our aim was to demonstrate its potential utility. Further targeted studies with larger study populations could confirm our diagnostic hypotheses and potentially compare them with reconstructions dedicated to ligament assessment, which we currently lack. Due to the small sample size, we did not perform any sample size calculations, and the statistical analysis was quite straightforward. Also, we could not perform specific subgroup analyses. Lastly, we did not evaluate alternative causes of ankle pain in patients without Achilles tendon lesions, which could be the subject of future studies. In conclusion, DECT proves to be an accurate method for diagnosing Achilles tendon injuries. Its accuracy was higher with more experienced readers and, naturally, increased with the severity of the studied lesion. In this context, DECT could be used as an alternative when MRI is unavailable, in cases with a high suspicion of structural injuries, or to rule out severe structural lesions in patients with nonspecific presentations.

## Figures and Tables

**Table 1:** The protocol with detailed imaging parameters for 3T ankle MRI.

Sequences	T1w FSE	T2w FSE	PDw FSE FS
Parameter			
Repetition time (ms)	515	6700	2895
Echo time (ms)	8	79	34
Matrix size	374 × 416	374 × 416	288 × 320
Bandwidth (Hz/pixel)	420	300	200
NSA	1	1	1
Echo train length	3	20	7
Slice thickness (mm)	2.5	2.5	2.5

**Table 2:** Demographic and clinical data of the participants presented as mean values with (range) and [standard deviation]. Clinical data are presented as the number of cases with (percentage).

Characteristics	No. of Participants (*n* = 22)
Age (y)	48.5 years (range 26–78 years) [12.3]
Number of men	12 (54.5.0%)
Number of women	10 (45.5%)
Normal tendon	10 (45.5%)
Tendon injuries	12 (54.5%)
Complete rupture	2 (9.0%)
Partial tear	8 (36.4%) Thickness 3.8 (range 1–8 mm) Length 16.2 (range 2–34 mm)
Tendinopathy	2 (9.0%)
Tendon’s thickness	
Control	6.5 mm (range 5.5 to 7 mm)
Injured tendons	10 mm (5.5 mm to 13 mm)

**Table 3:** Diagnostic performance of 3 readers at DECT versus MRI for diagnosing the presence of Achille’s tendon injuries (complete tear, partial tear, inflammatory changes).

DECT	R1	R2	R3
Overall assessment (accuracy)	90.9% (20/22)	86.4% (19/22)	81.8% (18/22)
	[80.6, 95.8]	[76.4, 93.8]	[0.72, 0.92]
Normal tendon	90% (9/10)	90% (9/10)	80% (8/10)
	[82.0, 96.5]	[82.0, 96.5]	[72.5, 86.5]
Partial tear	87.5% (7/8)	87.5% (7/8)	75% (6/8)
	[0.83, 0.92]	[0.83, 0.92]	[0.65, 0.85]
Complete tear	100% (2/2)	100% (2/2)	100% (2/2)
	[90.0, 1.0]	[90.0, 1.0]	[90.0, 1.0]
Inflammatory changes	100% (2/2)	50.0% (1/2)	100% (2/2)
	[90.0, 1.0]	[0.45, 0.55]	[90.0, 1.0]

Note: Percentages; fraction; 95% CIs in brackets. DECT = dual-energy CT.

**Table 4:** Inter-reader agreement according to dicotomic (0–1) Fleiss’ Kappa coefficient of concordance. MRI = magnetic resonance imaging; DECT = dual-energy CT.

	Fleiss’ Kappa	
	DECT	MRI *
Overall	0.88	0.92
Tears	0.94	0.97
Inflammatory changes	082	0.88
Thickness	0.96	0.95

* Referred to the comparison between 2 experienced MRI readers.

## Data Availability

Informed consent was obtained by all patients enrolled for this prospective study.
